# The effect of social capital on infectious disease-specific health literacy among community residents: the chain mediating effect of family health and psychological capital

**DOI:** 10.3389/fpubh.2026.1780476

**Published:** 2026-04-07

**Authors:** Huan Xu, Xixi Zhang, Qian Yang, Hanlu Du, Hongjuan Lang

**Affiliations:** 1 School of Nursing, Shaanxi University of Chinese Medicine, Xianyang, Shaanxi, China; 2 School of Nursing, Air Force Medical University, Xian, Shaanxi, China; 3 Xinjiang Military Command General Hospital, Urumqi, Xinjiang, China

**Keywords:** community residents, family health, infectious disease-specific health literacy, psychological capital, social capital

## Abstract

**Objective:**

This study aims to investigate how social capital affects infectious disease-specific health literacy among Chinese community residents, with a focus on the mediating roles of family health and psychological capital. The findings offer both theoretical insights and practical recommendations for enhancing infectious disease-specific health literacy among residents in a systematic manner.

**Methods:**

This cross-sectional study utilized convenience sampling to survey 415 residents from five communities in Xi’an, China, between September and October 2025. The survey collected data on sociodemographic characteristics, social capital, family health, psychological capital, and infectious disease health literacy. Data analysis was performed using SPSS 27.0 and AMOS 26.0.

**Results:**

Social capital had a significant direct positive effect on residents’ infectious disease-specific health literacy (*r* = 0.329, accounting for 35.45% of the total effect) and also exerted indirect effects through both independent and chained mediations involving family health and psychological capital. Specifically, the independent mediating effect of family health accounted for 38.90% of the total effect, while the effect of psychological capital contributed 17.03%. The combined chained mediating effect of both factors accounted for 8.62%. All hypotheses were confirmed.

**Conclusion:**

Social capital directly influences residents’ infectious disease-specific health literacy and indirectly affects it through the mediating pathways of family health and psychological capital. Therefore, implementing a comprehensive intervention strategy that “cultivates community social capital, optimizes family health functions, and enhances residents’ psychological capital” provides an effective approach to systematically improving infectious disease-specific health literacy among community residents and strengthening grassroots epidemic prevention networks.

## 1 Introduction

Infectious diseases are illnesses caused by various pathogens that can be transmitted between humans, animals, or both (1). The COVID-19 pandemic has highlighted the significant societal disruption that infectious diseases can cause. Although COVID-19 has been largely contained, infectious diseases like tuberculosis, AIDS, and malaria continue to affect many regions worldwide, causing widespread illness (2). In China, the incidence of infectious diseases remains high. The latest statistics indicate that 14.48 million new cases of legally notifiable infectious diseases were reported in 2024 (3). Therefore, appropriate measures must be implemented to reduce the incidence of infectious diseases.

Health literacy (HL) is strongly associated with population health behaviors, and low health literacy can lead to adverse health outcomes (4). In China, health literacy is categorized into six components: scientific health literacy, infectious disease-specific health literacy (IDSHL), chronic disease prevention and control literacy, safety and first aid literacy, basic medical literacy, and health information literacy (5). IDSHL refers to an individual’s ability to access, understand, evaluate, and apply health information and services in infectious disease-related situations (6). Recent reports show that only 29.26% of the Chinese population had IDSHL in 2024, the lowest among the six health literacy domains (5). Previous studies show a significant negative correlation between IDSHL levels and infectious disease incidence, with higher IDSHL levels linked to lower disease prevalence (7, 8). Therefore, enhancing IDSHL is a key strategy for reducing infectious disease incidence in populations. Investigating the factors that influence IDSHL will help develop targeted strategies to improve population-level IDSHL.

Social capital refers to intangible resources in society, such as trust, cooperation, and shared values. It is closely tied to individual qualities, social systems, and cultural traditions, positively influencing social and economic development (9). Numerous studies have emphasized the critical role of strong social capital for individuals, as it promotes both physical and mental well-being, while enhancing quality of life and satisfaction (10–12). Additionally, social capital is closely related to health literacy, with individuals possessing higher social capital often demonstrating greater health literacy (13, 14). The Social Determinants of Health (SDH) framework, as articulated by the World Health Organization (WHO), emphasizes that the primary determinants of population health and health inequities extend beyond healthcare quality and individual lifestyle differences. Instead, they are largely shaped by the comprehensive social, economic, political, and environmental conditions in which people are born, grow, live, work, and age (15). Social capital reflects the social environment in which residents live, indicating that the level of social capital individuals possess may directly affect their IDSHL status.

Family health refers to resources at the household level, arising from the interaction of each family member’s health, capabilities, behaviors, and personalities, as well as internal dynamics and external resources, including the family’s physical, social, emotional, economic, and medical environments (16). Family health significantly influences diverse populations. Strong family health can alleviate depressive symptoms in both young and older individuals (17, 18), reduce anxiety in working parents (19), improve frailty in preoperative gastric cancer patients (20), and enhance self-efficacy in chronic disease patients (21). Similarly, family health positively influences individual health literacy (22, 23). According to SDH theory, social capital as a key social resource provides families with material support, emotional assistance, and health information. This improves family functioning, health perceptions, and the collective ability to cope with illness. As the primary environment for individual health socialization, improved family health levels can also enhance residents’ IDSHL status.

Psychological capital refers to positive psychological states demonstrated by individuals during their development, consisting of four core elements: self-efficacy, optimism, hope, and resilience. These elements can be measured, developed, and enhanced, aiding individuals in coping with challenges and achieving success (24), Liao et al. (25) surveyed 341 residents and found significant correlations between residents’ psychological capital, social capital, and health-promoting behaviors, with psychological capital mediating the relationship between social capital and health behaviors. Leonti and Turliuc (26) also found a significant positive correlation between individuals’ psychological capital and health-related quality of life. According to positive psychological capital theory, residents’ psychological capital can be enhanced by favorable external factors, which in turn positively influence their health behaviors (24). Therefore, we propose that sufficient social capital provides individuals with physiological and psychological support, aiding residents in enhancing their psychological capital. Residents with high psychological capital typically demonstrate higher health literacy (27).

By integrating SDH and positive psychological capital theories, we conclude that residents’ social capital reflects their social, economic, political, and environmental conditions. Sufficient social capital provides families with abundant resources, offering material and emotional support to improve family health, transforming families into more resourceful and supportive units. As the fundamental and central social unit for residents, improved family health provides individuals with a stable and secure environment. This significantly nurtures the development of residents’ psychological capital. Ultimately, residents with high psychological capital exhibit the strongest motivation and ability to adopt healthy behaviors, resulting in higher IDSHL.

In summary, based on the SDH framework and positive psychological capital theory, this study investigates the pathways through which social capital influences residents’ IDSHL. It further examines the mediating effects of family health and psychological capital, providing theoretical support and guidance for improving IDSHL among Chinese residents. The study presents the following hypotheses:

H1: Social capital significantly influences Chinese residents’ IDSHL.

H2: Family health mediates the relationship between social capital and IDSHL in Chinese residents.

H3: Psychological capital mediates the relationship between social capital and IDSHL in Chinese residents.

H4: Social capital influences IDSHL in Chinese residents through a chain-mediated effect involving family health and psychological capital.

## 2 Methods

### 2.1 Study design and participants

This study is a cross-sectional investigation conducted in strict accordance with STROBE (Strengthening the Reporting of Observational Studies in Epidemiology) principles (28). Between September and October 2025, residents from five communities in Xi’an, China, were selected using convenience sampling and administered a questionnaire. Inclusion criteria were as follows: (1) age ≥18 years; (2) residence in the community for at least 6 months; (3) informed consent and voluntary participation. Exclusion criteria included: (1) cognitive impairment preventing independent completion of the questionnaire; (2) inability to provide valid contact information.

According to sampling calculation principles (29), the sample size should be 10–20 times the number of questionnaire dimensions, adjusted for a 15% attrition rate. Therefore, this study required a minimum of 253 residents, with 415 ultimately participating.

### 2.2 Instruments

#### 2.2.1 General information questionnaire

A self-designed survey was used, covering gender, age, marital status, education level, occupation, per capita monthly household income, self-rated health, and proactive learning about infectious disease knowledge.

#### 2.2.2 Social capital

Residents’ social capital was measured using the Social Capital Scale developed by Mujahid et al. (30). This scale has been widely used in China and has demonstrated good applicability (31, 32). The scale consists of two dimensions: social cohesion (4 items) and social interaction (5 items). Scoring was done using a 5-point Likert scale, with 1 indicating “strongly disagree” and 5 indicating “strongly agree.” Total scores ranged from 9 to 45, with higher scores indicating greater social capital. In this study, the Cronbach’s *α* coefficient for the scale was 0.91.

#### 2.2.3 Family health

Crandall et al. developed the Family Health Scale Short Form (33), Wang et al. translated the scale into Chinese and validated its reliability and validity within a Chinese population (34). The scale consists of four dimensions: Family Social and Emotional Health Processes (3 items), Family Healthy Lifestyle (2 items), Family Health Resources (3 items), and Family External Social Support (2 items). A 5-point Likert scale was used for scoring, where 1 denotes “strongly disagree” and 5 denotes “strongly agree.” The total score ranges from 10 to 50 points, with higher scores reflecting better family health. In this study, the scale demonstrated strong reliability and validity, with a Cronbach’s *α* coefficient of 0.87.

#### 2.2.4 Psychological capital

Residents’ psychological capital was assessed using the Psychological Capital Questionnaire 12 (PCQ-12) developed by Luthans et al. (24). This is a shortened version of the Psychological Capital Questionnaire 24 (PCQ-24), and has shown high reliability in Chinese populations (35). The scale consists of four dimensions: Self-Efficacy (3 items), Hope (3 items), Optimism (3 items), and Resilience (3 items). Scoring uses a 5-point Likert scale, where 1 denotes “Strongly Disagree” and 5 denotes “Strongly Agree.” The total score ranges from 12 to 60, with higher scores indicating greater psychological capital. In this study, the Cronbach’s *α* coefficient for this scale was 0.88.

#### 2.2.5 Infectious disease-specific health literacy

Tian et al. (36) developed the IDSHL scale for the Chinese population, which effectively measures residents’ infectious disease-specific health literacy. The scale assesses four dimensions: Infectious Disease-related knowledge and values (7 items), Infectious Disease prevention (7 items), Management or treatment of infectious diseases (4 items), and Identification of pathogens and infection sources (4 items). The total score ranges from 0 to 38.62 points, with higher scores reflecting greater IDSHL. The scale demonstrated strong validity in this study, with a Cronbach’s *α* coefficient of 0.85.

### 2.3 Data collection

This study was conducted using China’s online questionnaire platform, Wenjuanxing. We initially contacted the community neighborhood committee and obtained support from its administrators. Research team members, in collaboration with community service staff, recruited participants from within the community. Participants completed the questionnaire on their mobile phones by scanning a QR code. The questionnaire’s QR code was distributed through offline and online channels. Offline methods included postings on community bulletin boards and building entrances, while online distribution involved notifications via community WeChat groups and official resident committee accounts. The questionnaire included detailed explanations of its content and purpose. Participants could proceed only after completing the electronic informed consent form, and they could withdraw at any time during the process. To ensure data validity, two attention-check items were incorporated (e.g., “Please select ‘Strongly Disagree’ for this item”). Responses that failed these checks were classified as invalid and excluded from the analysis. Furthermore, responses completed in less than 10 min were excluded, as the duration was significantly shorter than the estimated 15–20 min. To prevent duplicate entries, each IP address was restricted to a single submission. After excluding those that failed to meet time requirements or exhibited patterned responses, 415 valid questionnaires remained, yielding a response rate of 92.2%.

### 2.4 Statistical methods

Data analysis was performed using SPSS 27.0. Sociodemographic characteristics were presented as frequencies and percentages. After the descriptive analysis, the Unmeasured Latent Method Factor (ULMF) approach was used to assess potential common method bias. Continuous variables, including social capital, family health, psychological capital, and IDSHL, followed normal distributions and are presented as means and standard deviations. Pearson correlation analysis was used to assess relationships between continuous variables.

Structural equation models were built using AMOS 26.0. Model validation was conducted using bootstrap sampling (5,000 repetitions), and mediation effects were tested with 95% confidence intervals (CI). A two-tailed *p*-value of <0.05 was considered statistically significant.

## 3 Results

### 3.1 Common method bias test

This study used the Unmeasured Latent Methodology Factor (ULMF) approach to assess common method bias. Results showed that after incorporating the latent methodology factor, changes in all fit indices (CFI, TLI, RMSEA, and SRMR) were less than 0.02. Furthermore, after controlling for the latent methodology factor, the significance and direction of all hypothesized substantive paths remained consistent. Therefore, no significant common method bias was identified in this study.

### 3.2 Discriminant validity

Discriminant validity was assessed using the Heterotrait–Monotrait Ratio (HTMT). The results showed that all HTMT values were below the 0.85 threshold, confirming strong discriminant validity among the latent variables.

### 3.3 Demographic characteristics of participants

A total of 415 community residents participated in the study. Of these, 211 were female (50.8%) and 204 were male (49.2%). Of the participants, 232 (52.9%) were married, and 41 (9.9%) held a bachelor’s degree or higher. Refer to Table 1 for details.

**Table 1:** Demographic characteristics of the participants (*N* = 415).

Variables	Categories	Number	*N* (%)
Total		415	100
Gender	Female	211	50.8
	Male	204	49.2
Age (years)	18 ~ 35	111	26.7
	36 ~ 50	143	34.5
	51 ~ 65	93	22.4
	>65	68	16.4
Marital status	Married	232	55.9
	Unmarried	174	41.9
	Divorced	9	2.2
Education level	Primary school or below	53	12.8
	Junior high school	108	26.0
	Senior high school or technical secondary school	139	33.5
	Junior college	74	17.8
	Bachelor’s degree or above	41	9.9
Occupation	Employed personnel	236	56.9
	Freelance	25	6.0
	Unemployed/Underemployed	29	7.0
	Student	26	6.2
	Retired	99	23.9
Household monthly income per capita (yuan)	<3,000	98	23.6
	3,000 ~ 5,000	165	39.8
	5,000 ~ 8,000	111	26.7
	>8,000	41	9.9
Self-health assessment	Poor	26	6.3
	Fair	277	66.7
	Good	112	27.0
Follow infectious disease-related information	Often	79	19.0
	Occasionally	185	44.6
	Rarely	125	30.1
	Never	26	6.3

### 3.4 Associations between social capital, family health, psychological capital, and IDSHL

The results are presented in Table 2. The mean scores for social capital, family health, psychological capital, and IDSHL were (33.92 ± 8.73), (39.69 ± 9.23), (46.18 ± 11.35), and (24.39 ± 9.22), respectively. Significant correlations were found between social capital, family health, psychological capital, and IDSHL. Specifically, social capital was positively correlated with IDSHL (*r* = 0.813, *p* < 0.01). Family health was positively correlated with both social capital (*r* = 0.757, *p* < 0.01) and IDSHL (*r* = 0.684, *p* < 0.01). Psychological capital was positively correlated with both social capital (*r* = 0.822, *p* < 0.01) and IDSHL (*r* = 0.805, *p* < 0.01). Family health was positively correlated with psychological capital (*r* = 0.662, *p* < 0.01).

**Table 2:** Descriptive statistics and correlations of social capital, family health, psychological capital, and infectious disease-specific health literacy (*N* = 415).

Variables	M	SD	1	2	3	4
Social capital	33.92	8.73	1.000			
Family health	39.69	9.23	0.757^**^	1.000		
Psychological capital	46.18	11.35	0.822^**^	0.662^**^	1.000	
Infectious disease-specific health literacy	24.39	9.22	0.813^**^	0.684^**^	0.805^**^	1.000

**p < 0.01.

### 3.5 Model fit

Table 3 lists the confirmatory factor analysis fit indices: *χ*^2^/df = 1.762, RMSEA = 0.043, SRMR = 0.020, IFI = 0.992, TLI = 0.990, and CFI = 0.992. All indices meet conventional thresholds, indicating that the measurement model exhibits excellent fit to the data.

**Table 3:** Fitness indexes of the confirmatory factor analysis.

Index	*χ*^2^/DF	RMSEA	SRMR	IFI	TLI	CFI
Criteria	1–3	<0.08	<0.08	>0.9	>0.9	>0.9
Actual value	1.762	0.043	0.020	0.992	0.990	0.992

*χ*^2^, chi-square; DF, degrees of freedom; RMSEA, root mean square error of approximation; SRMR, standardized root mean square residual; IFI, the incremental fit index; TLI, tucker-lewis index; CFI, comparative fit index.

Table 4 lists the structural equation model fit indices: *χ*^2^/df = 1.913, RMSEA = 0.047, AGFI = 0.934, IFI = 0.991, TLI = 0.988, and CFI = 0.991. All indices meet conventional thresholds, indicating that the integrated structural model exhibits excellent fit to the data.

**Table 4:** Fitness indexes of the structural equation model.

Index	*χ*^2^/DF	RMSEA	AGFI	IFI	TLI	CFI
Criteria	1–3	<0.08	>0.8	>0.9	>0.9	>0.9
Actual value	1.913	0.047	0.934	0.991	0.988	0.991

*χ*^2^, chi-square; DF, degrees of freedom; RMSEA, root mean square error of approximation; AGFI, adjusted goodness-of-fit index; IFI, the incremental fit index; TLI, tucker-lewis index; CFI, comparative fit index.

### 3.6 Mediation effect analysis

**Figure 1:**
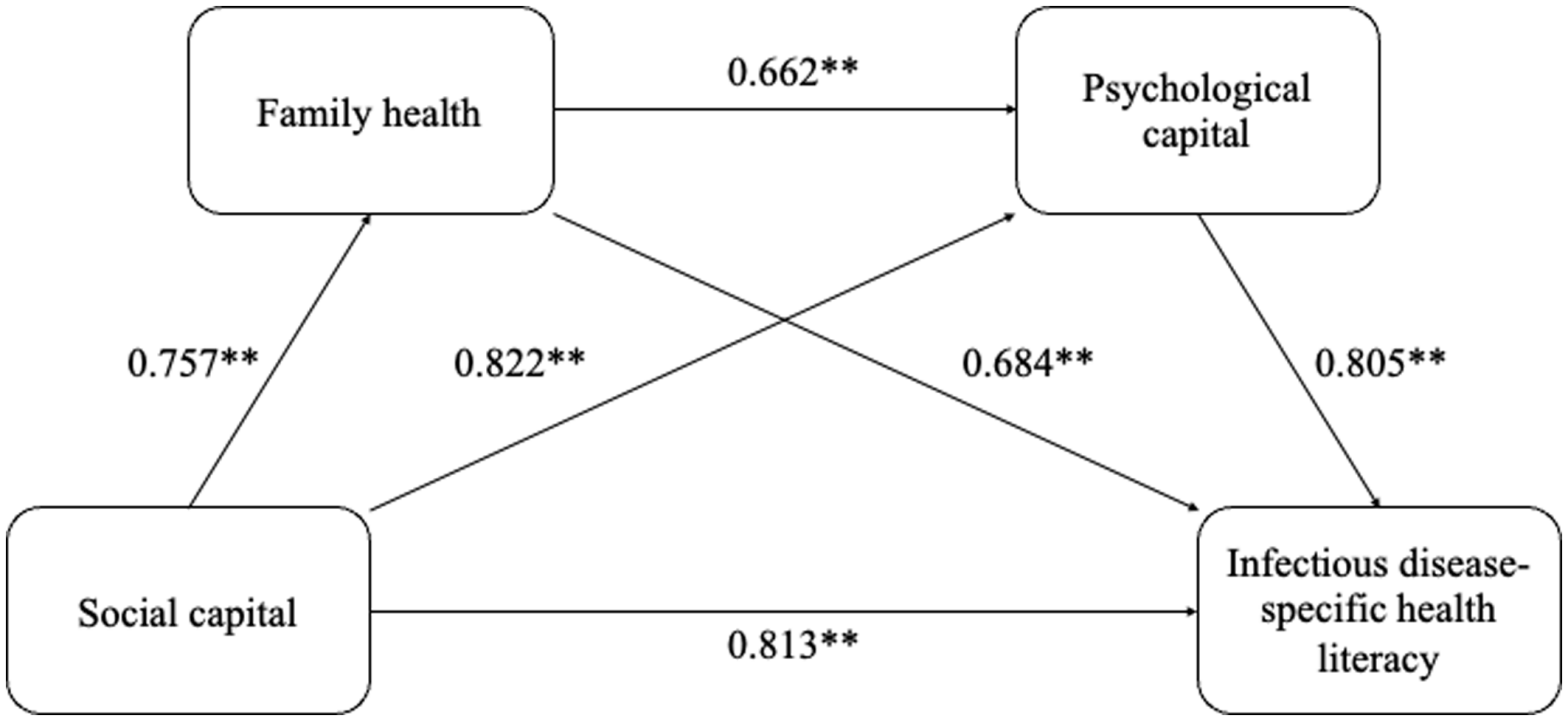
The chain-mediating model of social capital, family health, psychological capital, and infectious disease-specific health literacy in community residents; ^**^p < 0.01.

Figure 1, Table 5 display the results of the chain mediation model. We divided the effect of social capital on IDSHL into three components: total, direct, and indirect effects. The direct effect of social capital on IDSHL was 0.329, representing 35.45% of the total effect. Family health significantly mediates the relationship between social capital and IDSHL, with an effect size of 0.361, representing 38.90% of the total effect. Additionally, psychological capital significantly mediates this relationship, with an effect size of 0.158, representing 17.03% of the total effect. The chain mediation effect of family health and psychological capital was 0.080, representing 8.62% of the total effect. In summary, family health, psychological capital, and their combined influence contributed an indirect effect, representing 64.55% of the total effect of social capital on IDSHL.

**Table 5:** The standardized total, direct, and indirect effects of social capital on infectious disease-specific health literacy with family health and psychological capital (*N* = 415).

Model pathway	*β*	SE	95%CI		Percent (%)
			Lower	Upper	
Total effect	0.928	0.023	0.879	0.968	100
Direct effect	0.329	0.076	0.176	0.473	35.45
Social capital→ infectious disease-specific health literacy					
Indirect effect	0.599	0.063	0.484	0.732	64.55
Social capital→ family health→ infectious disease-specific health literacy	0.361	0.067	0.240	0.499	38.90
Social capital→ psychological capital→ infectious disease-specific health literacy	0.158	0.033	0.103	0.234	17.03
Social capital→ family health→ psychological capital→ infectious disease-specific health literacy	0.080	0.034	0.024	0.157	8.62

## 4 Discussion

This study examines the influence of social capital on residents’ IDSHL in Chinese communities, exploring its underlying mechanisms and pathways. Results show that social capital directly affects residents’ IDSHL and also influences it through independent and chained mediating effects of family health and psychological capital. This finding offers theoretical guidance and empirical support for the development of intervention programs aimed at enhancing residents’ IDSHL.

Our findings show that the average IDSHL score among Chinese residents is 24.39 (±9.22). Compared to previous studies, although Chinese residents’ IDSHL has improved in recent years, the pace of improvement remains relatively slow (36, 37). Additionally, Chen et al. (6) found that 65.3% of Chinese community residents wish to improve their IDSHL. Therefore, it is crucial to identify key factors influencing residents’ IDSHL and develop targeted intervention strategies. The social capital scores observed in this study (33.92 ± 8.73) exceeded those reported by Wang et al. (31). This discrepancy may be attributed to differences in participant characteristics; while Wang et al. (31) focused on clinical patients, the present study surveyed general community residents. Both family health (39.69 ± 9.23) and psychological capital (46.18 ± 11.35) were at moderately high levels, consistent with previous studies (23, 38, 39).

This study finds that social capital significantly positively influences residents’ IDSHL. Specifically, higher social capital among residents is associated with higher IDSHL. Therefore, Hypothesis 1 is supported. Social capital is particularly important for community residents. Social capital optimizes residents’ use of healthcare resources, promotes healthy behaviors, and enhances quality of life (40–42). Luo et al.’s (13) survey of 1,600 community residents found that during infectious disease outbreaks, social capital was closely associated with health literacy. Chen et al. (43) also found that social capital enhances health literacy, helping residents cultivate healthy lifestyle habits. Our findings validate the importance of social determinants in understanding the mechanisms influencing health literacy. Individuals with high social capital often access more resources through networks, trust, and norms, which elevates their health literacy. Therefore, enhancing residents’ IDSHL relies on strengthening community trust and mutual aid networks. Communities should organize health-themed activities focused on neighborhoods or interest groups, turning health behaviors into “new norms” that promote community identity. Establish volunteer-based health mutual aid groups to share health information regularly and address queries on community platforms, focusing on vulnerable groups, such as older adults living alone, ensuring that information and support reach every part of the network.

Our research shows that family health mediates the relationship between social capital and residents’ IDSHL. In other words, higher social capital is associated with better family health, which in turn leads to higher IDSHL. Hypothesis 2 is supported. Tschida et al.’s study confirms that high social capital encourages family members to engage in physical exercise, contributing to improved family health (44). A survey of 5,473 participants found a positive correlation between family health and health literacy, suggesting that family health is a key pathway to improving health literacy (22). Therefore, the first step is to promote regular health learning mechanisms within households. This involves encouraging family members to monitor authoritative epidemic prevention information from the Centers for Disease Control and Prevention (CDC) and community health centers, standardizing household health behaviors and creating personalized family plans. Second, leverage the emotional support of families to encourage positive health behaviors and offer gentle reminders for lapses. Use the emotional bonds within families to reduce resistance to behavioral change.

We found that psychological capital mediates the relationship between social capital and residents’ IDSHL, such that higher social capital is associated with greater psychological capital, which in turn leads to higher IDSHL. Hypothesis 3 is supported, consistent with previous research findings (25). Both social capital and psychological capital show strong correlations across different groups (45, 46). Additionally, individuals with high psychological capital often have higher health literacy (27, 47). Our results further confirm the applicability of positive psychological capital theory in explaining the mechanisms influencing residents’ IDSHL. When residents’ social capital increases, it enhances psychological capital, facilitating its improvement. Conversely, individuals with high psychological capital are more motivated and confident in learning infectious disease knowledge and implementing prevention measures. Therefore, the significant role of residents’ psychological capital in influencing IDSHL levels deserves attention. First, enhance residents’ health self-efficacy through community-based practical training in epidemic prevention and the sharing of successful prevention cases. Second, reduce pandemic-related anxiety through online and offline psychological support communities and expert Questions and Answers (Q&A) sessions, conveying scientific progress in prevention and positive messages to cultivate residents’ resilience and optimism in facing the pandemic.

Our findings also show a significant positive correlation between family health and psychological capital among residents, with social capital mediating IDSHL through a chain effect involving family health and psychological capital. Hypothesis 4 is supported. Family health is crucial for individual psychological well-being. A survey of 9,859 residents in China found that improving family health reduces the risk of emotional disorders (48). At the same time, robust family health enhances self-efficacy and psychological resilience among family members (21, 22). Our findings support the use of SDH and positive psychological capital theories in explaining the mechanisms influencing residents’ IDSHL. Residents’ social capital reflects their ability to access resources through social networks, trust, and norms within society. Higher social capital enables residents to secure better living conditions and use these advantages to improve family health. As the most fundamental social unit, healthy families enhance individuals’ self-efficacy in infectious disease prevention and control, fostering optimism, hope, and resilience during epidemics and prevention efforts. Additionally, enhanced psychological capital further elevates residents’ IDSHL. Therefore, alongside strengthening residents’ social capital, improving both family health and psychological capital can better enhance residents’ IDSHL.

## 5 Limitations

This study has several limitations. First, the cross-sectional design requires caution in interpreting the results, as it limits the ability to draw valid inferences about causal relationships between variables. Future research should incorporate longitudinal studies to address temporal sequence issues and recall bias while strengthening causal evidence for mediating mechanisms. Second, data collection relied on self-reporting, which may introduce bias. Future studies could enhance the credibility of conclusions by integrating multiple data sources, such as third-party reports and objective indicators. Third, the study focused on residents from only five communities in Xi’an, which limits the generalizability of the findings. Expanding the sample to include multi-center data could validate the applicability of the conclusions across different geographical contexts. Finally, this study examined only the mediating effects of family health and psychological capital. Future research could include additional variables to further elucidate the pathways through which social capital influences residents’ IDSHL.

## 6 Conclusion

Enhancing the IDSHL of community residents is crucial for safeguarding public health and reducing the risk of infectious disease incidence. This study, based on the SDH and positive psychological capital theories, examined the mechanisms through which social capital influences infectious disease health literacy. Results indicate that social capital significantly influences residents’ infectious disease health literacy, both directly and through independent and chained mediating effects of family health and psychological capital, validating the applicability of these theories in community health promotion research. These findings offer crucial guidance for developing multi-level, targeted strategies to enhance IDSHL. They clarify core intervention pathways: “cultivating community social capital, optimizing family health, and strengthening residents’ psychological capital” and offer robust theoretical foundations and practical guidance for systematically improving residents’ health literacy through a cohesive “community-family-individual” framework.

## Data Availability

The original contributions presented in the study are included in the article/supplementary material, further inquiries can be directed to the corresponding author.

## References

[1] Wang X Zhang X Chen S Shi K Cui W Shi F . Infectious disease-specific health literacy and its influencing factors: research results based on a cross-sectional design study carried out in Shandong Province's rural areas. Medicine (Baltimore). (2025) 104:e42084. doi: 10.1097/md.0000000000042084, 40193649 PMC11977720

[2] Rethinking approaches to overcome infectious disease. Nat Microbiol. (2025) 10:1791–2. doi: 10.1038/s41564-025-02093-6 40745045

[3] National Disease Control and Prevention Administration. (2025). 2024 National Statutory Infectious Diseases Situation Summary. Available online at: https://www.ndcpa.gov.cn/jbkzzx/c100016/common/content/content_1998217472895258624.html (Accessed December 13, 2025).

[4] Li Y Lv X Liang J Dong H Chen C. The development and progress of health literacy in China. Front Public Health. (2022) 10:1034907. doi: 10.3389/fpubh.2022.1034907, 36419995 PMC9676454

[5] National Health Commission of the People’s Republic of China. (2025). Monitoring of Health Literacy among Chinese Residents in 2024. Available online at: https://www.nhc.gov.cn/xcs/c100122/202501/18ecbeb9c42942bea9e0fced7a963299.shtml (Accessed December 13, 2025).

[6] Chen J Liu K Cheng Q Wang L. Modeling health literacy intentions: a structural equation analysis of community residents’ willingness to acquire infectious disease specific health literacy. BMC Public Health. (2025) 25:2734. doi: 10.1186/s12889-025-23842-6, 40796841 PMC12341289

[7] Or PP Wong BY Chung JW. To investigate the association between the health literacy and hand hygiene practices of the older adults to help them fight against infectious diseases in Hong Kong. Am J Infect Control. (2020) 48:485–9. doi: 10.1016/j.ajic.2019.12.021, 32037202 PMC7132680

[8] Niu Z Qin Z Hu P Wang T. Health beliefs, Trust in Media Sources, health literacy, and preventive behaviors among high-risk Chinese for COVID-19. Health Commun. (2022) 37:1004–12. doi: 10.1080/10410236.2021.1880684, 33557620

[9] Coleman JS. Social capital in the creation of human capital. Am J Sociol. (1988) 94:S95–S120. doi: 10.1086/228943

[10] Xiang J Xing H. The promotion mechanism of physical and mental health of the elderly in China: the impact of the digital divide and social capital. BMC Public Health. (2025) 25:2457. doi: 10.1186/s12889-025-23411-x, 40660167 PMC12261863

[11] Huang CC Xie X Tu Y Jiang X. Community engagement and life satisfaction amongst older adults in Chengdu, China: moderated mediation by social capital, gender and age. Australas J Ageing. (2025) 44:e70073. doi: 10.1111/ajag.70073, 40776890 PMC12332700

[12] Fu Y Zhang S Guo X Lu Z Sun X. Socioeconomic status and quality of life among older adults with hypertension in rural Shandong, China: a mediating effect of social capital. Front Public Health. (2023) 11:1248291. doi: 10.3389/fpubh.2023.1248291, 37927868 PMC10622776

[13] Luo Y Zhao H Chen H Xiao M. Association between cultural capital and health literacy during the COVID-19 pandemic among community residents in China: the mediating effect of social capital. Front Public Health. (2023) 11:1199941. doi: 10.3389/fpubh.2023.1199941, 38026294 PMC10647931

[14] Amoah PA Tang VMY Adjei M. Social capital as an instrument for health literacy promotion among community-dwelling older adults in Hong Kong. Glob Public Health. (2025) 20:2486433. doi: 10.1080/17441692.2025.2486433, 40194895

[15] Vakili F Nasiri M Jahanfar S Shishehgar S Mahmoodi Z Hamzehgardeshi Z . Social determinants of health and menopausal symptoms: path analysis using the WHO framework. BMC Public Health. (2025) 25:4304. doi: 10.1186/s12889-025-25612-w, 41444560 PMC12729258

[16] Weiss-Laxer NS Crandall A Okano L Riley AW. Building a Foundation for Family Health Measurement in National Surveys: a modified Delphi expert process. Matern Child Health J. (2020) 24:259–66. doi: 10.1007/s10995-019-02870-w, 31912378

[17] Zhang Z Liu W Zhang C Sun L. The relationship between family health, stress, and self-efficacy on depression among university students: a large-scale national cross-sectional study. Front Public Health. (2025) 13:1625269. doi: 10.3389/fpubh.2025.1625269, 40823223 PMC12350489

[18] Hao R Jin H Zuo J Wu Y Sun X Hu J. The multiple mediating effect of family health and perceived social support on depressive symptoms in older adults: a cross-sectional national survey in China. J Affect Disord. (2023) 327:348–54. doi: 10.1016/j.jad.2023.01.097, 36731543

[19] Su C Yang M Huang Q Yang M. Balancing act: exploring the impact of work-family conflict on anxiety among working parents with family health as a mediator. Front Psychol. (2025) 16:1531091. doi: 10.3389/fpsyg.2025.1531091, 40755533 PMC12314395

[20] Xin H He C Gu Y Ma X Xiang Z Gong J. The association between family health and frailty in preoperative patients with gastric cancers: the mediating role of health literacy and physical activity. Front Public Health. (2025) 13:1541688. doi: 10.3389/fpubh.2025.1541688, 40487517 PMC12140988

[21] Luo ZN Li K Chen AQ Qiu YC Yang XX Lin ZW . The influence of family health on self-efficacy in patients with chronic diseases: the mediating role of perceived social support and the moderating role of health literacy. BMC Public Health. (2024) 24:3398. doi: 10.1186/s12889-024-20906-x, 39673060 PMC11639113

[22] Wang YY Huang XC Yuan J Wu YB. Exploring the link between family health and health literacy among college students: the mediating role of psychological resilience. Healthcare. (2023) 11. doi: 10.3390/healthcare11131859, 37444692 PMC10341136

[23] Ma Y Huang L Tian H Liu H Yu H Li H . The impact of health literacy on health-promoting lifestyle among community residents: the chain-mediating role of family health and physical activity. Front Psych. (2024) 15:1487274. doi: 10.3389/fpsyt.2024.1487274, 39583751 PMC11582026

[24] Luthans F Avolio BJ Avey JB Norman SM. Positive psychological capital: measurement and relationship with performance and satisfaction. Pers Psychol. (2007) 60:541–72. doi: 10.1111/j.1744-6570.2007.00083.x

[25] Liao L Li Y Tian F Wu J Zhong J He T . The mediating role of psychological capital in health behaviors among elderly nursing home residents. Front Psychol. (2025) 16:1534124. doi: 10.3389/fpsyg.2025.1534124, 39911995 PMC11794268

[26] Leonti RM Turliuc MN. Better and healthier together? The mediation effect of positive psychological capital on the relationship between perceived social support and health-related quality of life among older adults. Int J Aging Hum Dev. (2025) 100:502–26. doi: 10.1177/00914150241268178, 39140286 PMC12069828

[27] Zou J Liu Y. Perceived stress, positive psychological capital and health literacy in patients with multiple chronic conditions: a structural equation modelling. J Clin Nurs. (2025) 34:1303–11. doi: 10.1111/jocn.17201, 38764212

[28] Wiehn E Ricci C Alvarez-Perea A Perkin MR Jones CJ Akdis C . Adherence to the strengthening the reporting of observational studies in epidemiology (STROBE) checklist in articles published in EAACI journals: a bibliographic study. Allergy. (2021) 76:3581–8. doi: 10.1111/all.14951, 34022062

[29] Kline RB. Principles and Practice of Structural Equation Modeling. New York: The Guilford Press (2016).

[30] Mujahid MS Diez Roux AV Morenoff JD Raghunathan T. Assessing the measurement properties of neighborhood scales: from psychometrics to ecometrics. Am J Epidemiol. (2007) 165:858–67. doi: 10.1093/aje/kwm040, 17329713

[31] Wang L Li J Dang Y Ma H Niu Y. Relationship between social capital and depressive symptoms among type 2 diabetes mellitus patients in Northwest China: a mediating role of sleep quality. Front Psych. (2021) 12:725197. doi: 10.3389/fpsyt.2021.725197, 34616319 PMC8488102

[32] Gao J Fu H Li J Jia Y. Association between social and built environments and leisure-time physical activity among Chinese older adults--a multilevel analysis. BMC Public Health. (2015) 15:1317. doi: 10.1186/s12889-015-2684-3, 26715531 PMC4696285

[33] Crandall A Weiss-Laxer NS Broadbent E Holmes EK Magnusson BM Okano L . The family health scale: reliability and validity of a short- and long-form. Front Public Health. (2020) 8:587125. doi: 10.3389/fpubh.2020.587125, 33330329 PMC7717993

[34] Wang F Wu Y Sun X Wang D Ming WK Sun X . Reliability and validity of the Chinese version of a short form of the family health scale. BMC Prim Care. (2022) 23:108. doi: 10.1186/s12875-022-01702-1, 35524178 PMC9077878

[35] Zhang J Rehman S Addas A Ahmad J. Influence of work-life balance on mental health among nurses: the mediating role of psychological capital and job satisfaction. Psychol Res Behav Manag. (2024) 17:4249–62. doi: 10.2147/prbm.S497305, 39679316 PMC11646404

[36] Tian X Di Z Cheng Y Ren X Chai Y Ding F . Study on the development of an infectious disease-specific health literacy scale in the Chinese population. BMJ Open. (2016) 6:e012039. doi: 10.1136/bmjopen-2016-012039, 27496240 PMC4985922

[37] Qin J Gong Y Hu R Lou Y Xuan X Wang P . Associations of infectious disease-specific, electronic, and general health literacy among high school students with prevalent health challenges: a cross-sectional study. Front Public Health. (2025) 13:1613375. doi: 10.3389/fpubh.2025.1613375, 40438059 PMC12117663

[38] Shi X Li J Ji X Wu Y Zang S. The mediating effects of self-efficacy, family health, and perceived stress on the relationship between perceived social support and eHealth literacy in nursing students: a structural equation model. BMC Nurs. (2024) 23:868. doi: 10.1186/s12912-024-02546-z, 39616339 PMC11607907

[39] Guerrero-Alcedo JM Espina-Romero LC. Bayesian analysis of psychological capital in peruvian university students: differences by sex and age. Heliyon. (2024) 10:e35370. doi: 10.1016/j.heliyon.2024.e35370, 39166071 PMC11334823

[40] Li Z Li D Li Z. Digital literacy, life satisfaction, social capital, and multidimensional health behaviors among middle-aged and older adults in rural China: a nationwide cross-sectional study. Digit Health. (2025) 11:20552076251374114. doi: 10.1177/20552076251374114, 41018519 PMC12464424

[41] Feldens T Seghieri C Fontana A Berta P. Mediating effects between social capital and health care utilization in Italy-a structural equation model analysis. Popul Health Metrics. (2025) 23:75. doi: 10.1186/s12963-025-00441-6, 41422066 PMC12750791

[42] Buja A Akhtar S. The association between social capital and quality of life in old adults: a systematic review and meta-analysis. Front Public Health. (2025) 13:1668696. doi: 10.3389/fpubh.2025.1668696, 41283054 PMC12629930

[43] Chen WL Zhang CG Cui ZY Wang JY Zhao J Wang JW . The impact of social capital on physical activity and nutrition in China: the mediating effect of health literacy. BMC Public Health. (2019) 19:1713. doi: 10.1186/s12889-019-8037-x, 31856789 PMC6924071

[44] Tschida L de Brito JN Sapkota S Fertig AR Trofholz A Berge JM. Social determinants of health and parent and child physical activity: a cross-sectional and longitudinal exploration among socioeconomically and racially and ethnically diverse families. Am J Health Promot. (2025) 39:890–901. doi: 10.1177/08901171251327452, 40105108 PMC13367073

[45] Zhao X Liu Q Zhang S Li T Hu B. The impact of psychological capital and social capital on residents’ mental health and happiness during COVID-19: evidence from China. Front Psychol. (2022) 13:962373. doi: 10.3389/fpsyg.2022.962373, 35923727 PMC9339779

[46] Cao Q Chen CF Hu HL Hsiao YC. Social capital and job performance: a moderated mediation model of organizational citizenship behaviors and psychological capital. Behav Sci (Basel). (2025) 15:714. doi: 10.3390/bs15060714, 40564496 PMC12189108

[47] Zhai S Shen C Zhang J Huang J. The impact of positive psychological capital on health literacy among college students: the mediating role of health behavior intention. Acta Psychol. (2025) 261:105919. doi: 10.1016/j.actpsy.2025.105919, 41265326

[48] Lai X Liu J Zhao Y Wu X Wu L He K . Interrelationships of family health with depression and self-efficacy among Chinese adults: a latent profile analysis and network analysis. J Affect Disord. (2025):120964. doi: 10.1016/j.jad.2025.120964 41443318

